# Adjunctive role of Q10 with *Ligilactobacillus salivarius*, and *Lactiplantibacillus plantarum* probiotic Bacteria on the HEp-2 cells viability and adhesion of Streptococcus mutans

**DOI:** 10.3389/fcimb.2023.1053230

**Published:** 2023-04-28

**Authors:** Zohreh Khodaii, Shayan Mardi, Parham Mardi, Mahboobeh Mehrabani Natanzi

**Affiliations:** ^1^Dietary Supplements and Probiotic Research Center, Alborz University of Medical Sciences, Karaj, Iran; ^2^Student Research Committee, Arak University of Medical Sciences, Arak, Iran; ^3^Student Research Committee, Alborz University of Medical Sciences, Karaj, Iran; ^4^Evidence-based Phytotherapy And Complementary Medicine Research Center, Alborz University of Medical Sciences, Karaj, Iran

**Keywords:** co-enzyme Q10, probiotics, periodontal disease, ligilactobacillus salivarius, lactiplantibacillus plantarum

## Abstract

**Objective:**

Various studies have indicated the application of Coenzyme Q10 and probiotic bacteria such as *Ligilactobacillus salivarius (L. salivarius)* and *Lactiplantibacillus plantarum (L. plantarum)* in combating periodontal disease. Considering the positive effect of these two on oral health, and the destructive effect of *S. mutans*, in this study, we investigate the outcomes of the administration of probiotics and Q10 on infected HEp-2 cell viability and *S. mutans* adhesion in different settings.

**Methods:**

A 3-week-old human epidermoid laryngeal (HEp-2) cell line was cultured and exposed to two different probiotics and 3 different doses of Q10 doses. Samples were contaminated by *S. mutans* immediately (therapeutic setting) and after 3 hours (preventive setting). Eventually, the viability of HEp-2 cells was investigated by MTT. Also, the number of adhered *S. mutans* was explored by direct and indirect adhesion assays.

**Results:**

L. plantarum and L. salivarius protect epithelial cells against *S. mutans* in both therapeutic and preventive settings, albeit not fully. In contrast, Q10 completely preserves the viability of infected Her HEp-2 cells at all concentrations. The effects of the coexistence of Q10 and probiotics were not quite equal, among which L. salivarius and 5 μg of Q10 form the best results. The microscopic adherence assay of *S. mutans* revealed that samples containing Q10 had significantly lower adhesion of probiotics and *S. mutans* to HEp-2 cells. Similarly, plates containing *L. salivarius* with *5*μg or *L. plantarum* with 1μg Q10 or sole presence of *L. salivarius* had the lowest *S. mutans* adherence among others. Also, *L. salivarius* with *5*μg Q10 had one of the highest probiotic adherences.

**Conclusion:**

In conclusion, co-administration of Q10 and probiotics especially in presence of *L. salivarius* with *5*μg Q10 could have remarkable effects on HEp-2 cell viability, *S. mutans*, and probiotic adherence. Nevertheless, our study, for the first time, showed that Q10 might have an anti-bacterial activity by suppressing the adhesion of tested bacteria to HEp-2 cells. This hypothesis, if correct, suggests that due to their different mechanisms, co-prescription of Q10 and probiotics may lead to better clinical responses, especially in the mentiond dose.

## 1 Background

The study of human microbiota is rapidly progressing; until now, probiotics have been widely used in intestinal disease treatment. However, recent studies have shown that the function of these cells in the oral cavity can also be helpful (Thaiss et al., 2016; Young et al., 2019). Different hypotheses have been proposed regarding the pathophysiology of these bacteria in preventing dental infections. One of the leading hypotheses states that these bacteria prevent the spread of infection by creating a physical and biochemical barrier against pathobionts-host attachment (Honda and Littman, 2016). Also, these bacteria strengthen the host’s immune system by creating a balance between inflammatory systems in the absence or presence of infection (Kilian, 2018). For example, studies conducted on oral streptococci have shown evidence of immune-modulatory function and reducing pro-inflammatory responses (Kilian, 2018; Silva et al., 2019; Young et al., 2019).

Considering probiotics’ benefits and limited side effects, they have been widely available to the public, especially as probiotic-enriched food, mouthwashes, toothpaste, and anti-bacterial gums (Ranadheera et al., 2010; Jose et al., 2013). Studies have shown that these methods prevent tooth decay by reducing bacterial density in the oral cavity and preventing respiratory infections, colds, and allergies (Caglar et al., 2005; Winkler et al., 2005).

Among hundreds of known probiotics, the impact of *Ligilactobacillus salivarius (L. salivarius)* and *Lactiplantibacillus plantarum (L. plantarum)* on oral health is highly discussed (Kucia et al., 2020; Nordström et al., 2021). These two are known as oral cavity normal microbiota, and studies have shown that the presence of these two probiotics could regulate the oral microbiota, especially *S. mutans*, and prevents periodontal disease. For example, San Miguel et al. showed that *L. salivarius* protects host tissues from damage caused by other immunostimulatory cells and products by reducing the innate immune response and the NF-kappaB function in epithelial cells (Cosseau et al., 2008). Also, the effect of *L. plantarum* on *S. mutans* gene expression suppresses glucan-related genes and eventually prevents biofilm formation (Lim et al., 2020).

Although investigating probiotics and their preventive-therapeutic outcomes still needs much research, the results show that using these bacteria alone or simultaneously can effectively treat or prevent oral infections (Chugh et al., 2020). Like probiotics, micronutrition, especially coenzyme Q10 (Q10), is wildly administered as a preventive or therapeutic agent in periodontal disease (Dahiya et al., 2022). Q10 is an intracellular antioxidant and one of the crucial components of the electron transport chain, which plays an essential role in ATP formation (Hargreaves et al., 2020). Studies have shown that increasing its concentration in the oral epithelium suppresses periodontal inflammation (Rasoolzadeh et al., 2022). Similar to probiotic-containing supplements, oral gels containing Q10 are available and prescribed for periodontitis treatment. Due to the human larynx epithelioma cancer (HEp-2) resemblance to human oral epithelial cells, this cell line has been used as a model of infection induction in this study. This study was designed and implemented to investigate the outcomes of the administration of probiotics and Q10 on infected HEp-2 cell viability and *S. mutans* adhesion in different settings.

## 2 Materials and methods

### 2.1 Bacterial strain and media

The *Ligilactobacillus salivarius* (DSM 20555; Germany) and  Lactiplantibacillus plantarum (PTCC 1896; Iran) were cultured in De man Rogosa Sharp (MRS) broth or on MRS agar plates (Merck Co. Germany) at 37°C under anaerobic conditions (in an anaerobic jar, Oxoid Ltd, UK) for 18-20 hours and prepared in a concentration of 10^9^ colony-forming units (CFU)/ml.

*S. mutans* (PTCC 1683; Iran) was purchased from the Persian Type Culture Collection (PTCC; Iran) and cultivated in mitis -salivarius -bacitracin (MSB) agar (Merck Co. Germany). The turbidity of bacterial suspension was adjusted to McFarland standard no. 1, equivalent to 10^9^ CFU/ml. Nutrient Broth medium (NB) was used as a diluent.

### 2.2 Cell culture

A 3-week-old human epidermoid laryngeal (HEp-2) cell line (ATCC^®^ CCL-23™) was used. Various studies have widely used these cells as oral epithelial cell models (Mack et al., 1999; Haeri et al., 2012; Khodaii et al., 2017). HEp-2 cells were cultured in a 75 cm^2^ flask. The culture media was Eagle’s Minimum Essential Medium (EMEM) (ATCC® 30-2003™), fetal calf serum (Lablech 4-101-500) 10%, and Penicillin 10,000 unit/ml & streptomycin 10,000 g/ml (GIBCO 15140-122) 1%. cells were incubated at 37°C in a humidified atmosphere of 5% CO2 and 95% air and the medium was replaced by a new one every two days.

To observe the growth characteristics of HEp-2 cells, suspension of cells containing 2*10^4^ cells/well was applied to 24-well plates. Cell number was counted every day for 6 days after plating. The doubling time of HEp-2 cells without any treatment was 47.6 h.

### 2.3 Addition of Q10 and probiotics

HEp-2 cells were seeded in 12 sets of 24-well plates at 2*10^4^ cells/cm2 and cultured in the mentioned medium. After 72 h of culture, cells were washed with dulbecco’s phosphate-buffered saline (DPBS) and cultured with an antibiotic-free culture medium. After 12 h cells were washed twice with DPBS and 1 mL of culture medium without antibiotics was added.

**Figure 1:**
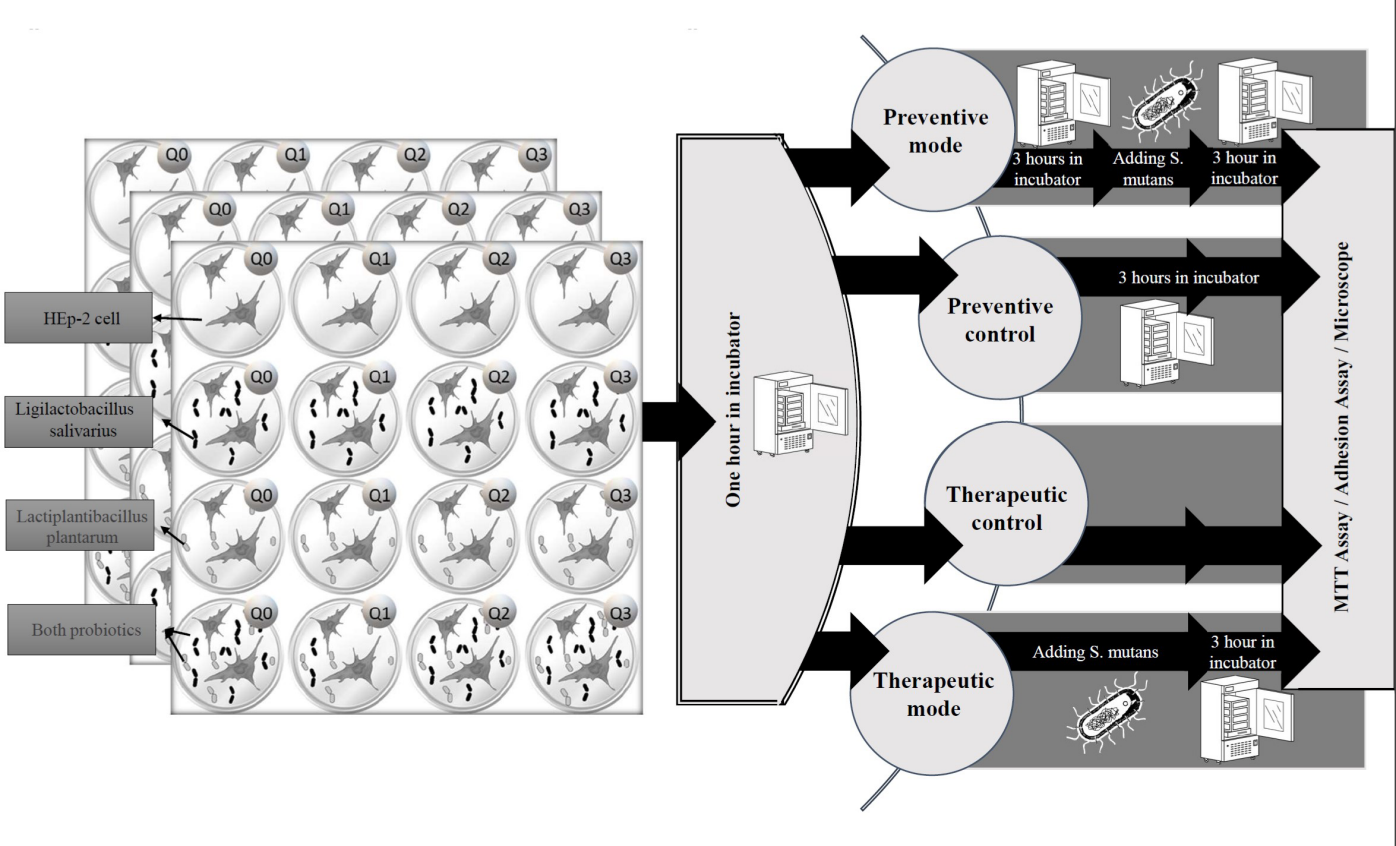
The experimental protocol. The study was conducted on treatment and preventive settings. In treatment mode, HEp-2 cells were infected by *S. mutans* immediately after administration of probiotics or Q10, and in preventive mode, the probiotics or Q10 were added three hours before infection.

Q10 was dissolved in distilled water and filtered. Plates’ first column did not receive any amount of Q10, in the second column, each well received 1μg Q10, and the third and fourth columns received 5μg and 10μg of Q10, respectively. The fifth and sixth columns remain empty. On the other hand, no probiotics were added to the first row but the second and third rows were treated with a 1.5 ml volume of *L. salivarius* and *L. plantarum* suspension at a concentration of 1*10^6^ cells/ml (as a total cell count), respectively. The fourth row carries both *L. salivarius* and *L. plantarum*. Finally, all plates were incubated for 1 hour (**Figure 1**).

### 2.4 Study protocol

To investigate the immediate and delayed effect of *S. mutans* on each combination, this experiment was established three times in four settings, containing: therapeutic mode, preventive mode, and their control. For therapeutic control adhesion, microscopic, and MTT assays were performed an hour after adding probiotics or Q10, but for preventive control, plates were incubated at the mentioned condition for 3 hours before any investigation. Each assay was carried out in duplicate and on two different occasions.

In preventive mode, three plates (each containing 16 full wells) were placed in the incubator for 3 hours at 37°C and then contaminated by live *S. mutans* and for therapeutic mode, an immediate infection with this bacteria was performed.

### 2.5 *S mutans* infection protocol

An overnight culture of *S. mutans* was centrifuged, and the growth medium was removed. The bacterial pellet was washed with DPBS and resuspended in the culture medium without antibiotics and anti-mycotic (D -MEM without P/S) to give about 10^6^ CFU/ml. Then one milliliter of mentioned medium with 10^6^ CFU *S. mutans*, with a multiplicity of infection (MOI) of 30 bacteria: 1 cell, were incubated with the pre-formed monolayer of HEp -2 cells for 3 hours at optimal conditions. The suspension with unattached *S. mutans* cells was removed, then the monolayers with adhered bacteria were washed three times with DPBS to remove unattached bacteria.

### 2.6 HEp-2 cell viability

HEp-2 cell Viability was measured by both microscopic and MTT assay. To conduct MTT colorimetric assay, a commercially available MTT assay kit (Sigma-Aldrich, UK) using a modified version of the Nga N method was used (Nga et al., 2020). MTT assay reagents were prepared as 5 mg/ml stock solutions in DPBS, sterilized by Millipore filtration, and stored in the dark. At appropriate time points, MTT stock solution (10% of total volume) was added to each well. After 3 hours of incubation at 37°C and 5% CO2, the reagent was aspirated and 200 µL of MTT solvent (Sigma-Aldrich, UK) was added to dissolve the formazan crystals. The solution was uniformly agitated on a shaker for 15 min to ensure proper dissolution. Spectrophotometer read the optical densities of formazan solutions at 570 nm on an ELISA plate reader (Tecan Sunrise, Tecan Austria) and background absorbance values ​​were measured at 650 nm. Recorded absorbance values ​​were assumed to be proportional to the number of viable cells in each sample well. Also, HEp-2 cell viability was investigated by counting alive HEp-2 cells in every plate under the microscope.

### 2.7 Indirect adhesion assay

The adhesion assay was also measured with both direct and indirect methods. In the indirect method, HEp-2 cell monolayers were washed three times with DPBS. HEp-2 cells with attached bacteria were solubilized by incubation with EDTA-Trypsin at 37° C. The number of adhered bacteria was determined by plating out of diluted bacterial suspension on blood agar or MRS agar plates depending on the bacterial strain. The developed colonies indicated adhered bacteria to the cell line (Ouwehand et al., 1999).

### 2.8 Direct adhesion assay

Also, the adherence assay was conducted using direct microscopy. After washing HEp-2 cell monolayers with DPBS, The remaining bacteria attached to the monolayer were stained with 1% [w/v] in water Crystal violet for 5 min and quantified using an inverted light microscope (Nikon Diaphot, x 400 magnification), according to Negri et al. (2010). The number of *S. mutans* and probiotics, adhering to 100 epithelial cells were counted microscopically at a magnification of A400 in 5 fields.

### 2.9 Statistical analysis

All the data is reported in the form of mean ± SD. The differences between other groups are measured by ANOVA and later with the Duncan test, and a p-value under 0.05 is considered significant.

## 3 Results

### 3.1 HEp-2 cell viability

#### 3.1.1 Effect of interventions and infection on normal HEp-2 cells

**Figure 2:**
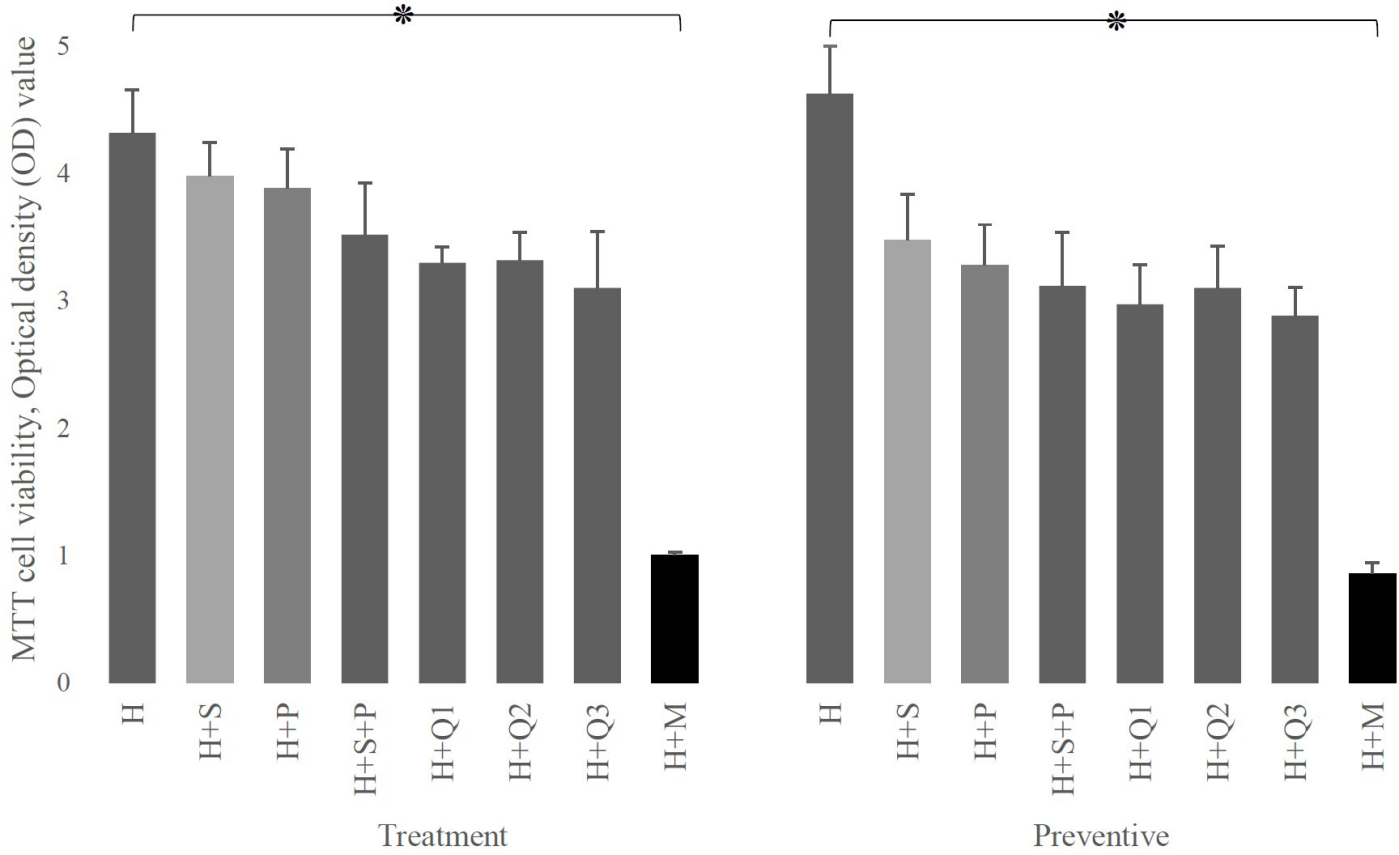
The effect of P (*L. plantarum*), S *(L. salivarius*), and M (*S. mutans)* on the viability of H (HEp-2 cell) in treatment and preventive mode. As shown, the presence of any of the substances used in this study (different doses of Q10 or probiotics) either immediately (therapeutic settings) or after 3 hours (preventive settings) by itself does not have a destructive effect on HEp-2 cells, while the presence of mutans strongly affects the survival of these cells. The values are the mean ± SD of these assays. Asterisk (*) indicates a significant difference (p-value<0.05) in the values when compared to the control.

Data showed that regardless of setting, the simultaneous growth of HEp-2 cells and defined interventions did not significantly differ from the growth of HEp-2 cells alone (**Figure 2**). Also, as demonstrated in **Figure 2**, there is a significant decrease in the HEp-2 survival rate after infection by *S. mutans* (*p-value*: 0.0001).

#### 3.1.2 The sole effect of probiotics or Q10 on infected HEp-2 cells

**Figure 3:**
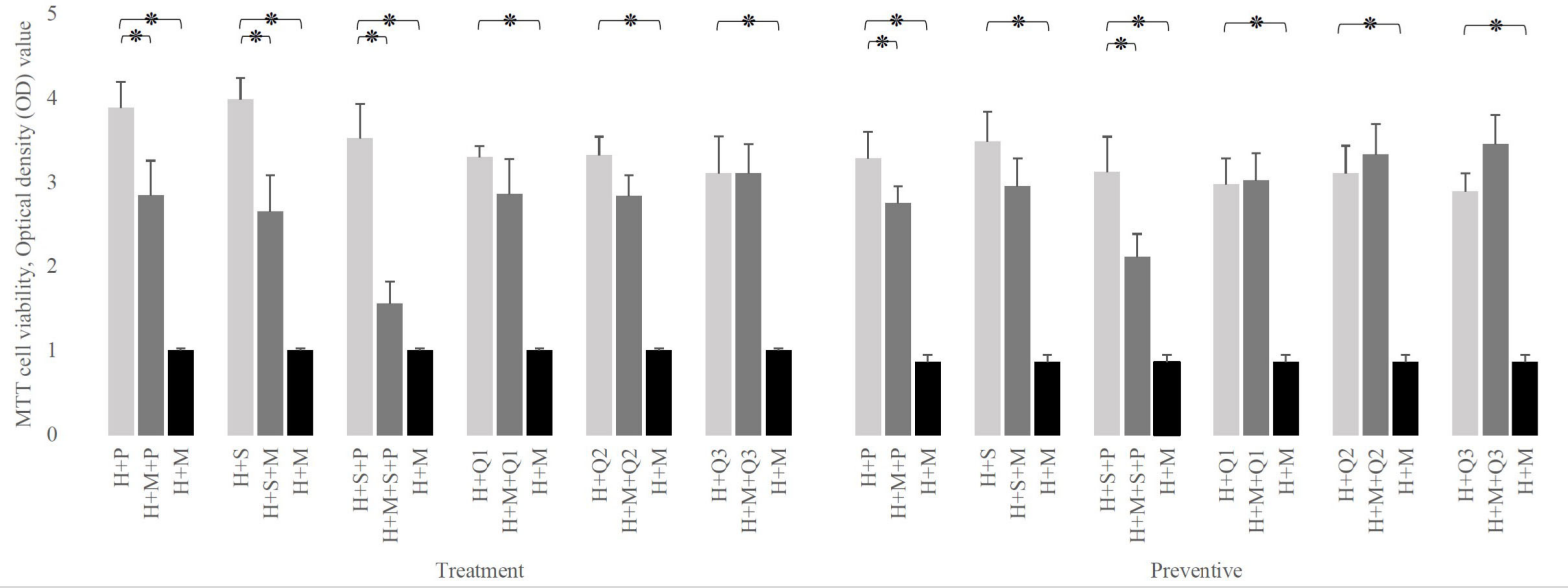
Comparison of H (HEp-2 cell) viability in the presence of P (*L. plantarum*), S (*L. salivarius*), and M (*S. mutans)* to the absence of one of them. The simulation’s presence of any doses of Q10 or L*. salivarius* preserves the viability of HEp-2 cells in both therapeutic and preventive settings. The values are the mean ± SD of these assays. Asterisk (*) indicates a significant difference (p-value<0.05) in the values when compared to the control.

As shown in **Figure 3**, even in the presence of probiotics, infection of HEp-2 cells with *S. mutans* leads to a significant reduction in HEp-2 cell survival rate, except for prevention with *L. salivarius* (*p-value:* 0.0652). Albeit this decrease in HEp-2 cell viability was not as much as their absence (*p-value* less than 0.001).

Also, **Figure 3** reveals that in the presence of Q10, at any doses and any setting, *S. mutans* infection does not affect the viability of HEp-2 cells (*p-value* > 0.05). Moreover, the simultaneous addition of Q10 and *S. mutans* to infected HEp-2 cells increased their survival rates from 1.007± 0.02 (*S. mutans* without Q10) to 2.859 ± 0.412 at 1 μg, 2.838 ± 0.241 at 5 μg, and 3.102± 0.352 at 10 μg (*p-value:* 0.0001). Similar data have been seen in preventive mode.

#### 3.1.3 Effect of probiotics and Q10 co-addition on infected HEp-2 cells

**Figure 4:**
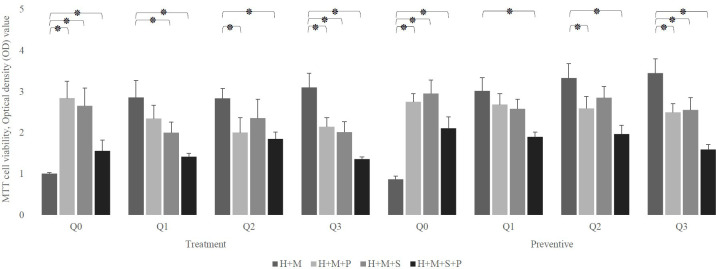
The effect of probiotics (P, *L. plantarum*; S, *L. salivarius*) on the viability of infected H (HEp-2 cell) treated with Q10 (Q0, absence Q10; Q1, 1μg Q10; Q2, 5μg Q10; and Q3, 10μg Q10) in the treatment and preventive mode. The existence of probiotics in the absence of Q10 preserves the viability of HEp-2 cells compared to the absence of probiotics. On the other hand, the addition of probiotics to a Q10-contained environment decreases the infected HEp-2 cells’ viability, regardless of setting, except for the coexistence of 1μg Q10 with *L. plantarum* and 5μg Q10 with *L. salivarius* in both settings and prevention with 1μg Q10 with *L. salivarius.* The values are the mean ± SD of these assays. Asterisk (*) indicates a significant difference (p-value<0.05) in the values when compared to the control.

As shown in **Figure 4**, regardless of the experimental setting, the presence of probiotics in a Q10-free well significantly increases the viability of infected HEp-2 cells compared to their absence. in the therapeutic setting, this increment was from 1.007± 0.02 to 2.843 ± 0.412 (in the presence of *L. plantarum*), 2.654 ± 0.432 (in the presence of *L. salivarius*), and 1.557± 0.265 (in presence of both probiotics). Similarly, in the preventive setting, it raises from 0.865± 0.08 to 2.749± 0.204, 2.954± 0.328, and 2.111± 0.275, in the presence of *L. plantarum, L. salivarius*, and both, respectively.

Also, **Figure 4**, demonstrates that in both therapeutic and preventive modes, the addition of probiotics to Q10-contained wells reduces the viability of infected HEp-2 cells except in 5 conditions: the prevention and treatment of infected HEp-2 cells with 1 μg of Q10 and *L. plantarum (p-value:*0.2397 and 0.1625, respectively), the prevention and treatment of infected HEp-2 cells with 5 μg of Q10 and *L. salivarius (p-value:* 0.1364 and 0.1844, respectively) and prevention of *S. mutans* infection with 1 μg of Q10 and *L. salivarius (p-value*: 0.1276).

**Figure 5:**
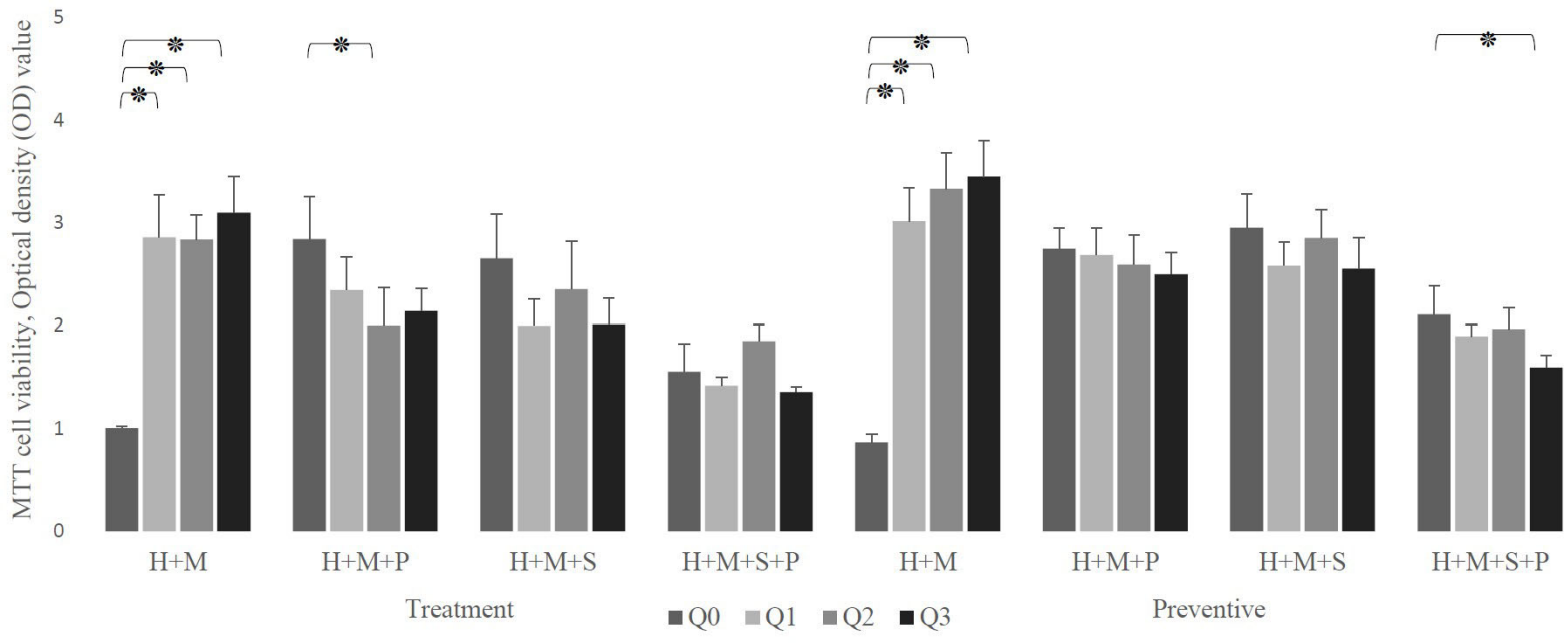
The effect of the presence of Q10 on the viability of infected H (HEp-2 cell) treated with probiotics (P*, L. plantarum*; S, *L. salivarius*) in the treatment and preventive mode. The presence of Q10 at all doses leads to the prevention and treatment of HEp-2 cells. Also, co-treatment of these cells with Q10 and probiotics at some doses and prevention at all doses preserves their viability. The values are the mean ± SD of these assays. Asterisk (*) indicates a significant difference (p-value<0.05) in the values when compared to the control. (Q0, absence Q10; Q1, 1μg Q10; Q2, 5μg Q10; and Q3, 10μg Q10).

Despite the fact that all concentrations of Q10 preserve the viability of infected in absence of probiotics, (*p-value* less than 0.0001) our preventive data show that the co-addition of Q10 to probiotic-rich environments not only does not change the viability of infected cells, but also the copresence of 10 μg of Q10 with *L. plantarum* and *L. salivarius* in comparison to the coexistence of both probiotics decrease the viability of infected HEp-2 cells from 2.111± 0.275 to 1.596± 0.12 (p-value: 0.0410). Nevertheless, the therapeutic data demonstrated that the simultaneous addition of Q10 and probiotics to *S. mutans*-infected HEp-2 cells does not change the viability compared to the sole existence of probiotics unless in the presence of 5 μg of Q10 with *L. plantarum (p-value*=0.0578) (**Figure 5**). Also, the results of the microscopic counting of live HEp-2 are summarized in **Table 1**.

**Table 1:** The percent of live HEp-2 cells in the microscopic assay.

	Q0	Q1	Q2	Q3
HEp-2 + *L. plantarum*	89	79	76	76
HEp-2 + *L. plantarum + S. mutans*	65.4	54.3	46.4	49.6
HEp-2 + *L. salivarius*	92	74	79	71
HEp-2 + *L. salivarius + S. mutans*	61.4	46.4	54.5	46.7

100 HEp-2 cells in 5 fields at a magnification of A400 have been counted, and the percent of viable cells is reported in this table. Q0 (without Co Q10), Q1 (1μg of Co Q10), Q2 (5μg of Co Q10), and Q3 (10μg of Co Q10).

### 3.2 Adhesion to HEp-2 cells

#### 3.2.1 *S. mutans* adherence

Counting the mean number of adhered bacteria to 100 HEp-2 cells, revealed that aside from the effect of probiotics, the sole presence of Q10 in the HEp-2 cell culture medium decreased the number of HEp-2 cell-adhered *S. mutans* from 676 (in absence of any probiotics or Q10) to 633, 440, and 241 in presence of 1, 5, and 10 μg of Q10.

Also, the addition of probiotics without Q10 has similar results, On average, in absence of any substance, 676 bacteria adhered to each HEp-2 cell, but the presence of *L. salivarius* and *L. plantarum* decrease it to 328 and 454 bacteria per cell.

**Figure 6:**
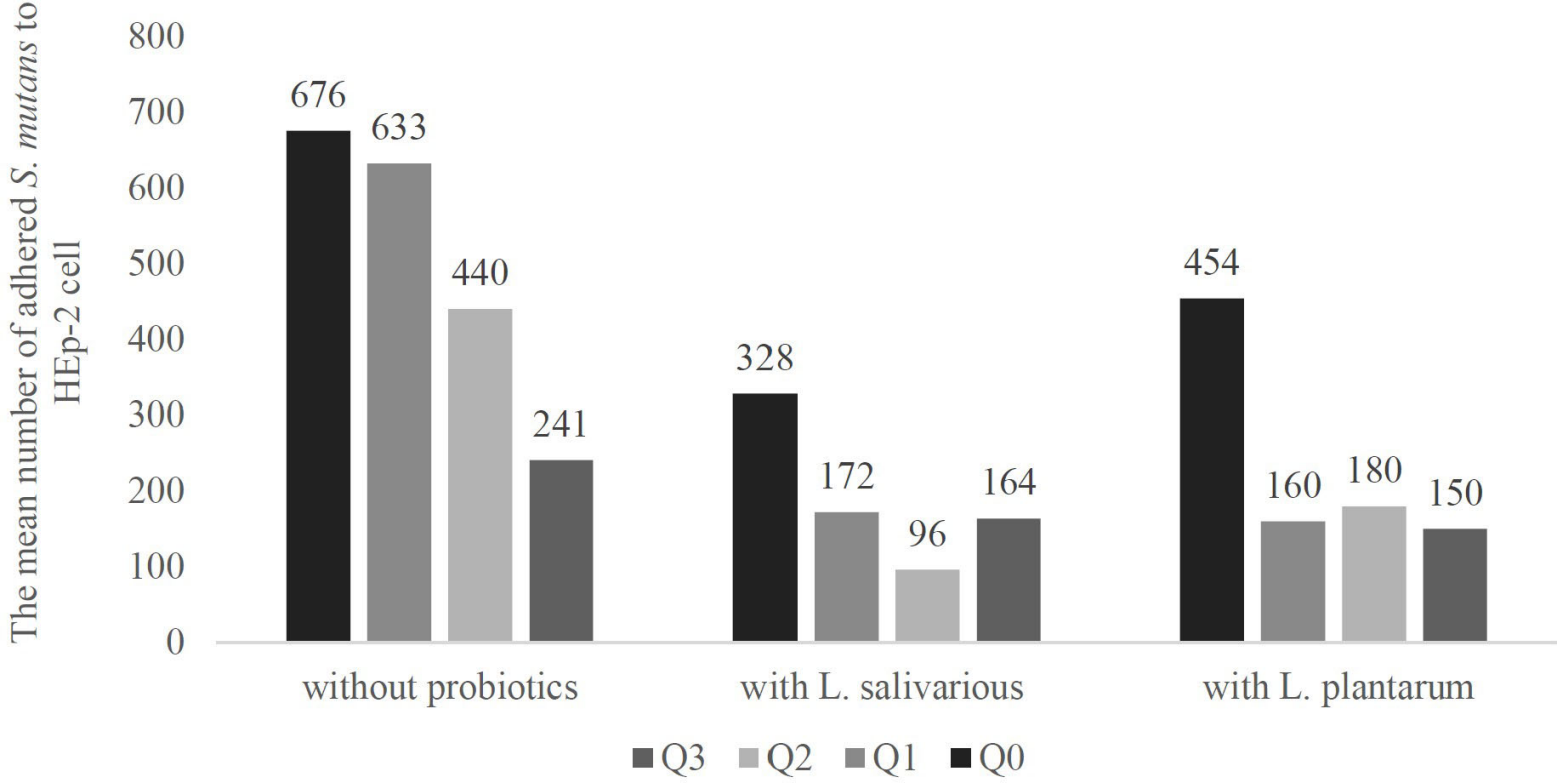
The adherence of *S. mutans* to in presence and absence of probiotics and Q10. S. mutants adhesion is an important step in the pathophysiology of various diseases, and higher adherence may lead to lower viability of HEp-2 cells and other dental complications. The adherence was measured using a microscope and observing 100 Hep-2 cells in 5 fields. The results indicate the mean number of adhered *S. mutans*. Q0, absence Q10; Q1, 1μg Q10; Q2, 5μg Q10; and Q3, 10μg Q10.

Our data showed that the co-addition of Q10 and probiotics compare to their sole existence vastly reduces the number of adhered *S. mutans.* The best results have been shown in the presence of *L. plantarum* and 1μg of Q10 (160 bacteria per HEp-2 cell), *L. plantarum* and 10μg of Q10 (150 bacteria per HEp-2 cell), and *L. salivarius* and 5μg of Q10 (96 bacteria per HEp-2 cell) (**Figure 6**).

The indirect method results (**Table 2**) showed that preseance of *L. plantarum* decreases the number of cell-attached *S. mutans* from 10^5^ CFU to 10^4^ CFU. However, the cocontaminant addition of probiotics with any dose of Q10 has extensive impact. The top results were seen in preseance of *L. salivarius* with 5 μg of Q10 (10^2^ CFU).

**Table 2:** *S. mutans* adherence.

	Q0	Q1	Q2	Q3
HEp-2 + *S. mutans*	10^5^			
HEp-2 + *L. salivarius* + *S. mutans*	10^5^	10^3^	10^2^	10^3^
HEp-2 + *L. plantarum* + *S. mutans*	10^4^	5x10^3^	10^3^	10^3^

The number of adhered S. mutans was determined by plating out of diluted bacterial suspension on Blood agar or MRS agar plates depending on the bacterial strain; the developed colonies indicated adhered bacteria to the cell line. Results indicate the CFU/ml of S. mutans in mentioned samples. Q0(without Co Q10), Q1 (1μg of Co Q10), Q2 (5μg of Co Q10), and Q3 (10μg of Co Q10).

#### 3.2.2 Probiotic adherence

**Table 3**, demonstrating the mean number of probiotic bacteria adhering to each epithelial cell, showed that on one hand, *L. salivarius* has a higher adherence rate than *L. plantarum* (198 vs. 140), and on the other hand, the adherence of probiotics to HEp-2 cells decreases in the presence of *S. mutans*. This reduction was from 140 to 120 in the presence of *L. plantarum* and from 198 to 124 in the existence of *L. salivarius*. As a matter of fact, in the sole presence of *S. mutans* the adherence rate of *L. salivarius* was almost the same as *L. plantarum.*

**Table 3:** Probiotic adherence.

	Q0	Q1	Q2	Q3
HEp-2 + *L. plantarum*	140	128	94	94
HEp-2 + *L. plantarum* + *S. mutans*	120	108	100	118
HEp-2 + *L. salivarius*	198	93	120	80
HEp-2 + *L. salivarius* + *S. mutans*	124	105	122	110

The number of probiotic bacteria adhering to 100 epithelial cells was counted microscopically at a magnification of A400 in 5 fields. Each assay was carried out in duplicate and on two different occasions. The results indicate the mean number of adhered probiotics. Q0 (without Co Q10), Q1 (1μg of Co Q10), Q2 (5μg of Co Q10), and Q3 (10μg of Co Q10).

Also, our data revealed that Q10 decreases the probiotics adherence in all concentrations. In absence of Q10, an average of 140 *L. plantarum* bacteria were attached to each HEp-2 cell, but adding 1, 5, and 10 μg Q10 reduce it to 128, 94, and 94 bacteria, respectively. Likewise, 198 *L. salivarius* bacteria adhered to each epithelial cell, and the addition of 1, 5, and 10 μg of Q10 decrease it to 93, 120, and 80 cells.

As expected, the co-presence of Q10 and *S. mutans* compared to the sole presence of *S. mutans* lowers the number of adhered probiotics. However, the co-addition of *L. salivarius* and 5μg Q10, *L. plantarum*, and 10 μg Q10 almost preserve the probiotic adhesion compared to the absence of Q10.

## 4 Discussion

Our current *in vitro* study reveals that *L. plantarum* and *L. salivarius* protect epithelial cells against *S. mutans* in both therapeutic and preventive settings, although not completely. In contrast, Q10, at all concentrations, entirely preserves the viability of infected HEp-2 cells. The effect of the co-presence of Q10 and probiotics was contrasting. Among them, *L. salivarius* and 5μg Q10 form the best results. The viability of infected HEp-2 cells entirely preserves in the presence of this combination in both therapeutic and preventive settings. Also, in their presence, we witnessed the lowest *S. mutans* adherence and complete prevention of *S. mutans’* effect in reducing the number of attached probiotics.

Our data agree with numerous *in vitro* and *in vivo* studies that suggest using one of these two probiotics against *S. mutans* (Caglar et al., 2005; Bonifait et al., 2009; Ogawa et al., 2011; Dodoo et al., 2020). Probiotic bacteria combat periodontal disease with different mechanisms. First of all, they affect both oral microbiota and immune responses; in this regard, a study by Mehrabani et al. showed that using probiotics as a dietary supplement, local treatment, or therapeutic alternative to antibiotic treatment is a promising way to boost the host immune system and decrease pathobionts adherence (Mehrabani Natanzi et al., 2018). Second, some probiotics can create a biofilm that replaces biofilm-growing bacteria such as *S. mutans*. Studies have suggested different probiotics with this effect, but *L. plantarum* and *L. salivarius* have had the most promising impacts on *S. mutans* and related diseases such as halitosis, periodontitis, and plaque. For instance, Lim et al. showed that *L. plantarum* could inhibit biofilm formation and glucan-related gene expression levels in *S. mutans*. Therefore it can be used as a biofilm inhibitor and an oral probiotic in functional foods (Lim et al., 2020). Also, Ogawa et al. demonstrated that *L. salivarius* inhibits biofilm formation by streptococci in the *in vitro* assay (Ogawa et al., 2011). Third, it has been suggested that these probiotics and *S. mutans* begin to compete for adhesions to HEp-2 cells, and as a result of this competition, fewer *S. mutans* can attach to target cells, leading to a significant reduction in the pathogenicity of these bacteria (Keller et al., 2011; Lee et al., 2019). In support of this hypothesis, our microscopic study clearly showed that increasing *L. plantarum* and *L. salivarius* adhesion leads to a lower adhesion rate of *S. mutans*; thus, HEp-2 cells’ survival rate improves. Nowadays, this is widely used in transforming and preserving normal microbiota.

Although Q10 is known as an intracellular antioxidant, studies have shown that local Q10 therapy is promising. In this regard, a double-blind study conducted by Matsumura et al. reported that oral hygiene combined with Q10 therapy could improve treatment results in certain patients with periodontitis (Matsumura et al., 1973). Also, Hanioka et al. observed that patients who received topical Q10 with subgingival debridement showed significant improvements in the plaque index and gingival index compared to patients who received only subgingival debridement (Hanioka et al., 1994). Similarly, Manthena et al. noted a significant reduction in gingival inflammation in the Q10 group compared to patients who received only scaling and root planning (Manthena et al., 2015).

However, the story does not end here; studies revealed that the Q10 activity is not limited to treatment but also may play a role in the etiology of periodontal disease. For example, Studies by Littaru, Henson, and Nakamura support that periodontal disease is significantly associated with Q10 deficiency (Littarru et al., 1971; Nakamura et al., 1974; Hansen et al., 1976). Likewise, our study showed that HEp-2 cell viability increased in the presence of different concentrations of Q10. Also, HEp-2 cells exposed to Q10 3 hours before infection had higher viability.

Different studies suggest various mechanisms for this co-enzyme in treating periodontal disease, including stimulating the immune system, increasing oxygen supply, reducing inflammation, or acting as an antioxidant on HEp-2 cells. For example, Hanioka et al. suggested that inflamed cells need higher oxygenation, so the administration of Q10 improves oxygen utilization and, thus, free radicals in inflamed gingival tissue. They concluded that the antioxidant activity of Q10 is the main mechanism of tissue healing (Hanioka et al., 1994). In contrast, Denny et al. assessed the antioxidant and anti-inflammatory effects of Q10 in 10 non-smoking periodontally healthy volunteers. They also demonstrated reductions in gingival bleeding after 28 days of supplementation but found no changes in gingival crevicular fluid total antioxidant capacity, indicating that the potential utility of Q10 may be independent of its antioxidant activity (Denny et al., 1999).

The anti-bacterial efficacy of this co-enzyme has been repeatedly reported in numerous studies (Nakamura et al., 1974; Wilkinson et al., 1975). For instance, McRee et al. report that oral administration of Q10 significantly reduced subgingival bacteria (MCREE et al., 1993); also, Block et al. found that 25 mg/kg of Q10 protects mice from some experimental bacterial infections (Block et al., 1978). These *in vivo* studies suggest that Q10 administration stimulates the immune system and suppresses bacterial growth. However, as our study was *in vitro*, This explanation is not just inadequate.

As mentioned above, the main results of our MTT assay were quite like the previous studies. However, we conducted the microscopic and adhesion assay to gain a better insight. These tests manifest a noteworthy observation that adding Q10 in the presence of probiotics decreases probiotics’ adhesion to HEp-2 cells (**Figure 6**), which is probably the reason for the reduced survival rate of infected HEp-2 after the simultaneous addition of probiotics and Q10 compared to each one alone. Also, we observed that Q10 alone decreases the adhesion of *S. mutans* to HEp-2 cells. Co-administration of Q10 and probiotics had a similar result. As shown in **Figure 4**, the simultaneous presence of this co-enzyme and any probiotics leads to a decrease in *S. mutans* adhesion. On the other hand, **Table 3** shows that the presence of Q10 has a negative impact on the adhesion of probiotics to HEp-2 cells.

As a matter of fact, our data illustrated that the presence of Q10 decreases the adhesion of all bacteria to HEp-2 cells, regardless of their pathogenicity and, obviously, the immune system. To explain this observation, we suggest a previously unmentioned mechanism that Q10s’ cell protection is not only for its antioxidant or immunomodulation activity but also Q10 may inhibit bacterial adhesion, which leads to a bacterial reduction in the *in vitro* and probably *in vivo* environment.

The effect of Q10 on cell wall stability has been illustrated in previous studies, mostly involving LDL biosynthesis; for instance, Eriksson et al. illustrated that Q10 stabilizes the cell membranes by its lipid ordering and condensing activity (Eriksson et al., 2018). Although tracing the exact mechanism demands extensive scrutiny, a review of involved pathways suggests the role of phosphatidylinositol-4,5-bisphosphate 3-kinase catalytic subunit alpha/beta/delta (PI3K). Tsai et al. demonstrated that Q10 suppressed the oxLDL-induced pathways, which affect Rho-associated protein kinase 2, and eventually PI3K, a key enzyme in focal adhesion.

Another significant point of this study is the improvement of L. salivarius adhesion and reduction of S. mutans adhesion to HEp-2 cells in the presence of 5μg of Q10. This probably shows that 5μg of Q10 is a suiTable concentration that prevents S. mutans adhesion as much as possible and, at the same time, has a minor negative impact on L. salivarius adhesion.

Despite the significant results, this study had some limitations that should be investigated in further studies, such as conducting qPCR on functional genes involved in adhesion or viability, investigating other concentrations of probiotics, and exploring the effect of Q10, *L. plantarum*, and *L. salivarius* on the proliferation of HEp-2 cells.

In conclusion, this *in vitro* study showed that the presence of *L. plantarum*, *L. salivarius*, and Q10 can significantly decrease the adhesion rate of *S. mutans* and improve the survival rate of infected HEp-2 cells in both therapeutic and preventive settings. Although the survival rates improved in the simultaneous presence of the two probiotics or Q10 with either, this improvement was not as significant as the presence of either of these substances alone. Our study suggests that they affect the survival of HEp-2 cells through very different mechanisms. As a result, there is a possibility that the simultaneous use of probiotics and Q10 in the oral environment will improve oral and dental health, respectively, through the improvement of oral microbiota and the overall reduction of bacterial adhesion. As a result, further investigations are required to ascertain the clinical efficacy of the co-administration of Q10 and these probiotics.

## Data availability statement

The raw data supporting the conclusions of this article will be made available by the authors, without undue reservation.

## Author contributions

SM drafted the manuscript and participated in experiments. PM conducted the statistical analysis, participated in structural editing, and revised the manuscript critically. ZK and MM equally supervised the project and resaved the manuscript. All authors contributed to the article and approved the submitted version.
